# Development and Utilization of Multifunctional Polymeric Scaffolds for the Regulation of Physical Cellular Microenvironments

**DOI:** 10.3390/polym13223880

**Published:** 2021-11-10

**Authors:** Youyi Tai, Aihik Banerjee, Robyn Goodrich, Lu Jin, Jin Nam

**Affiliations:** Department of Bioengineering, University of California, Riverside, CA 92521, USA; ytai001@ucr.edu (Y.T.); abane021@ucr.edu (A.B.); rgood002@ucr.edu (R.G.); ljin041@ucr.edu (L.J.)

**Keywords:** polymeric scaffold, multifunctional, physical stimuli, tissue engineering

## Abstract

Polymeric biomaterials exhibit excellent physicochemical characteristics as a scaffold for cell and tissue engineering applications. Chemical modification of the polymers has been the primary mode of functionalization to enhance biocompatibility and regulate cellular behaviors such as cell adhesion, proliferation, differentiation, and maturation. Due to the complexity of the in vivo cellular microenvironments, however, chemical functionalization alone is usually insufficient to develop functionally mature cells/tissues. Therefore, the multifunctional polymeric scaffolds that enable electrical, mechanical, and/or magnetic stimulation to the cells, have gained research interest in the past decade. Such multifunctional scaffolds are often combined with exogenous stimuli to further enhance the tissue and cell behaviors by dynamically controlling the microenvironments of the cells. Significantly improved cell proliferation and differentiation, as well as tissue functionalities, are frequently observed by applying extrinsic physical stimuli on functional polymeric scaffold systems. In this regard, the present paper discusses the current state-of-the-art functionalized polymeric scaffolds, with an emphasis on electrospun fibers, that modulate the physical cell niche to direct cellular behaviors and subsequent functional tissue development. We will also highlight the incorporation of the extrinsic stimuli to augment or activate the functionalized polymeric scaffold system to dynamically stimulate the cells.

## 1. Introduction

Over the past decade, advances in polymer science and engineering have led the progress of the tissue engineering field by providing solutions for innovative materials/structures to guide cellular behaviors. Typical tissue engineering strategies utilize scaffolds as a synthetic alternative for the natural extracellular matrix (ECM) to temporally support the cells, which require a 3D microenvironment resembling the in vivo conditions to develop a tissue with an appropriate structure and function. Polymers, both naturally derived and synthetic, have gained increased interest in the structural materials of tissue engineering scaffolds due to many advantages. These include the broad spectrum of biocompatible polymeric materials that can be used as tissue and cell culture platforms, the flexibility of the polymers that can be fabricated into various shapes with desired morphological features such as pores and their interconnectivity conducive to cell in-growth, and the existing mature synthesis technologies that enable the polymeric scaffolds to be easily and reproducibly produced. Hydrogel is the most commonly used polymeric biomaterials in tissue engineering due to its unique structural similarities to the native ECM [1].

Hydrogel is a 3D network of either physically or chemically cross-linked polymer chains that hold a large number of water molecules. Such a flexible structure of the hydrogel yields its control over shape, porosity, and surface morphology, providing a versatile platform for tissue engineering applications, including cell culture scaffolds, tissue barriers, and drug delivery vehicles [1]. More recently, the use of hydrogel, together with the 3D printing technique, provides a means to create engineered tissues composed of multiple phenotypic cells to form a tissue-like 3D structure [2]. Despite the great potential of hydrogel in tissue engineering, limitations such as relatively poor mechanical properties and scalability are significant challenges that need to be further addressed. Electrospinning has been utilized as one of the most employed scaffold synthesizing techniques for tissue engineering polymeric scaffolds [3].

When a high-voltage electric field is applied between a polymer droplet and a collector, the polymer droplet forms a cone shape, known as the Taylor cone, that ejects a jet of the polymer solution. The electrostatic repulsion and the rapid solvent evaporation will then separate the solution and create nano- or microfibers, which are attracted to and deposited onto the collector. Such fibrous structure formed by electrospinning resembles native ECM, supporting cell growth, differentiation, and maturation [4].

**Figure 1:**
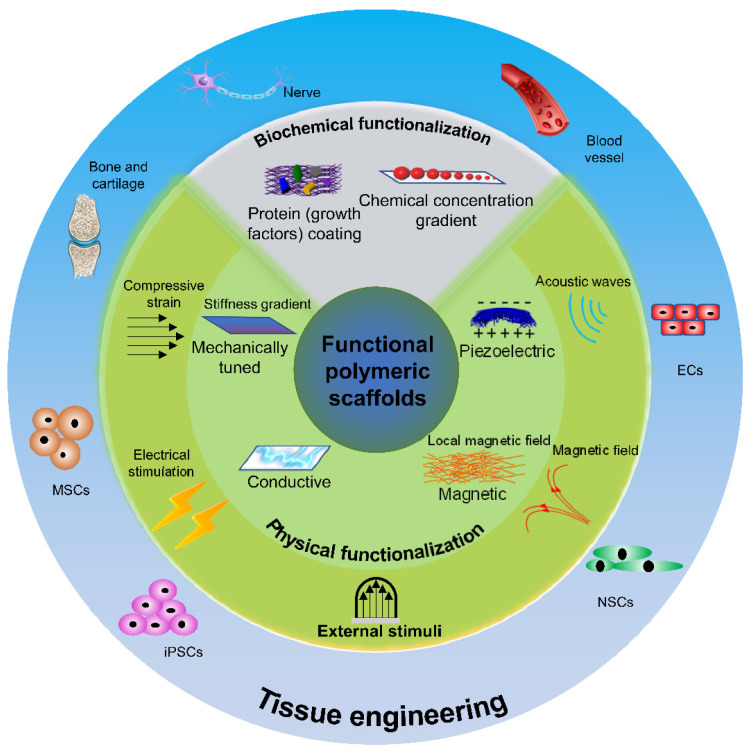
A schematic diagram of functional polymeric scaffolds and their applications in tissue and cell engineering. Abbreviations: endothelial cells (ECs), neural stem cells (NSCs), induced pluripotent stem cells (iPSCs), mesenchymal stem cells (MSCs).

Besides appropriate structural support, there are several properties that need to be taken into consideration when designing polymeric tissue scaffolds, including; (1) low cytotoxicity of polymers and their breakdown products; (2) good biocompatibility with low immunogenicity to reduce inflammatory responses after the implantation; (3) an appropriate rate of biodegradability designed for a specific tissue and its anatomical location; (4) high cell adhesion properties for the tissue morphogenesis of adherent cell types; (5) capability to provide appropriate chemical and physical microenvironment to the cells. To meet all these requirements, especially furnishing an adequate microenvironment for the cells, the scaffolds need to provide more than simple structural support by presenting various physicochemical cell niches. The most common and well-studied method is chemical functionalization, including polymer surface modification and biochemical delivery (Figure 1).

Surface coating can be easily achieved by physical adsorption or chemical conjugation of functional molecules to various natural and synthetic polymers, such as chitosan, collagen, polyvinylidene fluoride (PVDF), poly(ε-caprolactone) (PCL), and poly(L-lactic acid) (PLLA) [5,6,7]. Surface-coated polymers have numerous improved properties, including better biocompatibility, enhanced cell adhesion, control over cell selectivity and adhesion sites, improved cell proliferation, and enhanced cell differentiation to specific phenotypes [8,9,10,11,12]. In addition, a controlled release of biochemicals has been incorporated into polymeric scaffolds to modulate certain cellular behaviors, for example, the use of vascular endothelial growth factor (VEGF) concentration gradient within Matrigel to regulate endothelial cell migration [13].

Due to the complexity of cellular microenvironments in the native tissues, however, chemical modification is usually insufficient to fully develop functionalized tissues in vitro. In this regard, the control over physical microenvironments, including electrical, mechanical, and magnetic factors, has gained significant interest since they have been recently shown to crucially influence cellular behaviors, such as migration, proliferation, differentiation, and maturation, as well as to enhance tissue regeneration in bone, nerve, and blood vessels (Figure 1). Unlike biochemicals, in which their release is limited by initial loading, physical factors provide unlimited opportunity to stimulate the cells properly. As such, polymers with tunable stiffness have been investigated to examine the biomechanical environment-induced cellular behaviors, while conductive and piezoelectric polymers have been used to stimulate excitable tissues and cells [14,15,16,17,18,19]. In addition, recent studies have examined the effects of various extrinsic physical cues such as electrical stimulation, mechanical stimulation, and magnetic stimulation, on the behaviors of different tissues and cells cultured on functional polymeric scaffolds [20,21,22]. With an appropriate magnitude of each physical cue, such stimulation has been shown to enhance various cellular and tissue behaviors, including cell proliferation, cell migration, osteogenesis, neurogenesis, and angiogenesis.

In recent years, many excellent review articles discussed various aspects of polymeric scaffolds including synthesis, structuring, chemical modification as well as their clinical applications [23,24,25,26]. In this article, different polymeric scaffolds specifically developed for manipulating the physical microenvironments of the cells, are discussed. In addition, we summarize the recent research advances that utilized extrinsic stimuli, including electrical stimulation, mechanical stimulation, magnetic stimulation, or the combination of them, to further enhance the functionality of polymeric scaffold systems. Finally, we list and discuss the challenges and future directions regarding the use of multi-functional polymeric scaffolds in tissue engineering applications.

## 2. Conductive Polymeric Scaffolds

Electrical signals are ubiquitous in the physiological system where endogenous electric fields play a vital role in biological processes ranging from early embryonic development to tissue regeneration [27,28,29,30,31]. Ion concentration gradients across membranes are responsible for generating membrane potentials and conducting signals along biological membranes [32,33]. Endogenous electric fields have been shown to influence a variety of cellular processes such as chemotaxis, migration, proliferation, and differentiation of cells in addition to cell division, intracellular communication, neuronal activities, mechano-transduction, ion transport, bone, and epithelial healing [34,35,36,37,38]. Exogenous electrical stimulation positively influences the function and behavior of electroactive tissues such as nerve, muscle, and bone [39,40]. Studies on the impact of electrical fields on tissues date back to the 1960s when researchers demonstrated the effect of electrical stimulation on bone formation [41]. The effects of electrical signals in the wound healing process [40], or in vitro cellular behaviors such as migration, cytoskeletal organization, and alignment of neural, vascular endothelial, cardiofibroblasts, and myoblasts have already been well characterized [39,42].

It was also demonstrated how electric cues enhanced various regenerative cellular activities such as neurite outgrowth in nerve cells and enhanced collagen production and calcification in bone cells [42]. Based on such promising research outcomes, the therapeutic potential of electrical stimulation has been tested for accelerated wound healing, deep brain stimulation, tissue regeneration, improved musculoskeletal conditions, and recovery of bone fractures [43]. Therefore, external devices or electrodes are employed to apply physiologically safe electric currents, which underlines the importance of controlling the electrical characteristics of tissue engineering scaffolds for tissue regeneration (Table 1).

Electrically conductive polymers (CPs) are a class of novel materials that enable the direct application of electrical and electrochemical stimuli to tissues and cells [56], as listed in Table 1. Extensive research efforts are being undertaken regarding the application of CPs for biomedical applications such as bioactuators, biosensing, drug delivery, and bioimaging [57,58]. There are two major approaches to fabricate electrically conductive polymeric scaffolds; one utilizes the incorporation of conductive materials like carbon nanotubes into a non-conductive polymer matrix while the other mainly focuses on utilizing intrinsically conductive polymer materials. Carbon nanotubes (CNTs), either single-walled (SWNT) or multiwalled (MWNT), and graphene have been frequently used in tissue engineering [59,60,61]. Kabiri et al. investigated stem cells’ proliferation and neural differentiation on aligned electrospun PLLA scaffolds, loaded with either SWNT or MWNT. The addition of CNTs imparted conductivity to the scaffolds and guided mouse embryonic stem cells for neural differentiation, as evident from the expression of mature neuronal markers [44].

Crowder et al. demonstrated the functionality of an electrospun PCL scaffold embedded with CNTs to improve the cardiac differentiation of MSCs [47], which exhibited enhanced elongated rod-like morphology in 3D culture. Martinelli et al. showed that CNT-based scaffolds assist cardiomyocyte growth and proliferation by the electrophysiologic regulation of the gene expression pattern. They showed that ventricular myocytes cultured on MWNT scaffolds show enhanced survival and proliferation [45,46]. Li et al. demonstrated that the poly(N-isopropylacrylamide) (PNIPAm)/SWNTs hydrogel showed considerably higher cell attachment and proliferation of encapsulated stem cells, as compared to pure PNIPAm hydrogel. Furthermore, when acting as a vehicle for intramyocardial delivery of stem cells after myocardial infarction, the PNIPAm/SWNTs gel considerably assisted the hybridization of cultured cells in infarct myocardium and increased their therapeutic efficacies [62].

Moreover, Kharaziha et al. fabricated hard and flexible hybrid CNT-containing poly (glycerol sebacate)/Gelatin nanofibrous scaffolds with improved electrical properties which facilitated better beating action from cardiomyocytes [63]. The gelatin-methacrylate hydrogel containing CNT was shown to promote myocardial cell attachment, organization, and cell-cell communication by Shin et al. [64]., while SWNTs blended into collagen scaffolds promoted cardiomyocyte adhesion and proliferation, which was shown by Sun et al. [48]. Despite these phenomenological observations showing anabolic effects of CNT-based conductive materials for electroactive cells/tissues, the safety and biocompatibility of CNTs for in vivo applications are debatable [59,60,61].

Due to their unique electrical properties, polypyrrole (PPy), polyaniline (PANi), and poly(3, 4-ethylenedioxythiophene) (PEDOT) are the common standalone CPs that are frequently utilized in the field of tissue engineering [18,65]. PPy is one of the most commonly used CPs in tissue engineering due to its high electrical conductivity, superior processability, ease of surface modification, and biocompatibility [66,67]. PPy has been used as an in vitro cell culture substrate, and its in vivo performance has also been assessed in animal models. For example, PPy was electropolymerized in xanthan hydrogels, resulting in enhanced cell proliferation due to the favorable material characteristics such as hydrophobicity and surface roughness from electrical charging [49]. Another research group fabricated an electroactive scaffold consisting of magnesium (Mg), PPy-block-PCL, and poly (lactic-co-glycolic acid) (PLGA) as a core–shell-frame model for tissue engineering with enhanced biodegradability and biocompatibility [50]. Additionally, a conductive biodegradable scaffold based on PPy nanoparticles and poly(lactic acid) (PLA) was designed using emulsion polymerization, which maintained a physiologically relevant electric current for extended durations in addition to supporting enhanced fibroblast growth [51].

For neural tissues, Huang et al. fabricated a biodegradable conductive composite of PPy and chitosan to apply external electrical stimulation to Schwann cells, which revealed that low voltages (100 mV/mm) induce beneficial effects on cellular activities but higher voltages (300–1000 mV/mm) cause detrimental effects. Neurite outgrowth was also shown to be highly elevated by electrical stimulation applied through the conductive scaffold in vivo; the production of nerve growth factor (NGF) and brain-derived neurotrophic factor (BDNF) from Schwann cells was considerably elevated by electrical stimulation, which might have contributed to enhanced neurite outgrowth and nerve regeneration [52].

Another research group seeded osteoblasts-like Saos-2 cells on an electroactive layer made of PLA and bioactivated PPy using heparin (PPy/HE) [53]. The effect of electrical stimulation via the conductive polymer on the mineralization of osteoblast showed elevated osteoblast growth and adhesion, resulting in considerably higher calcium and phosphate concentration in the mineral precipitation with similar characteristic features to hydroxyapatite (HA), a native bone mineral. Electrical stimulation also upregulated the expression of the osteoblasts-specific markers runt related transcription factor 2 (Runx-2), alkaline phosphatase (AP), bone morphogenetic protein 2 (BMP2), and osteocalcin, demonstrating the anabolic effects of electrical stimulation on bone cells.

PANi is another CP that offers ease of synthesis, biocompatibility, low cost, as well as natural antibacterial properties [56]. PANi is the only CP whose electrical properties can be adjusted properly via charge-transfer doping and/or protonation. Quite a few studies have delineated the impact of PANi substrates on cellular activities [68,69,70]. Similar to PPy, blending PANi with biodegradable polymers like PLA or other natural polymers has been shown to enhance biodegradability while exhibiting enhanced electrical conductivity [40,68]. Li et al. have outlined the feasibility of electroconductive polymers in myocardial tissue engineering by showing that the nanofibrous scaffolds made of gelatin and PANi, as a conductive substrate, supported rat cardiac myoblasts proliferation [54]. Wang et al. synthesized nanofiber yarn/hydrogel core–shell scaffolds to mimic skeletal muscles, which resulted in the enhanced induction of 3D cellular alignment and the subsequent formation of elongated myotube. An aligned core–shell nanofiber was fabricated by electrospinning the combination of PCL/PANi/Silk where the 3D structure enhanced the nutrient exchange and provided the proper milieu for better myoblast alignment and myoblast differentiation [71]. In addition to the utilization of conductive polymers in static conditions, further improved cellular behaviors were observed when an external electrical stimulation was applied. For example, electrical stimulation along electrospun conductive nanofibers of PANi/PLLA showed elevated cell proliferation and neurite outgrowth compared to PANi/PLLA scaffolds that were not subjected to electrical stimulation [40].

PEDOT has been considered as an alternative to PPy due to its greater resistance to oxidation and higher conductivity. In vitro toxicity and biocompatibility tests have shown that PEDOT is cytocompatible [56,72]. PEDOT-coated PLA scaffolds have been shown to possess adequate conductivity to relay electrical stimulation to cells [73]. PEDOT-coated fibers demonstrated greater hydrophilicity, thermal stability, and lower glass transition temperature in comparison to the pure PLA fiber while PLLA/PEDOT scaffolds have been shown to support cell migration, adhesion, and proliferation [55]. Crosslinked PEDOT:polystyrene sulfonate (PEDOT:PSS) was used to culture neural stem cells (NSCs) under 100 Hz-pulsed DC electrical stimulation (1 V with 10 ms pulses), and it was shown that the electrical stimulation induced the differentiation of NSCs towards a greater number of neurons with longer neurite. This was one of the first studies in which the PEDOT:PSS combination was used to extend human NSCs through the implementation of pulsed signals, directing their differentiation to neurons and promoting longer neurites [74].

The potential of conductive polymers in tissue engineering is significant because the electrical regulation of cellular activities is essential for the regeneration of injured tissues. However, there are certain obstacles when CPs are employed in tissue engineering. The glaring shortcomings of the available systems are poor polymer–cell interactions, relatively low biocompatibility of by-products, poor solubility, and processability, as well as independently uncontrollable mechanical properties. The inability of CPs to degrade at an appropriate rate is one of the greatest constraints for tissue engineering usages. In vivo persistence of CPs for a long time may trigger inflammatory reactions and the requirement for a second surgical process. The synthesis of materials with both electroactive and degradable attributes is extremely desirable which, however, remains a challenge. There are ongoing efforts to address such a challenge by new materials and different synthesis methods for obtaining scaffolds that are both biodegradable and electrically conductive [18,65].

## 3. Mechanically Tuned Polymeric Scaffolds

Tissues and cells in vivo constantly experience evolving mechanical microenvironments depending on the anatomical location and their developmental stage. Numerous studies have found that physical cues, including morphology, topography, availability of adhesion sites, and mechanical properties of substrates, play a crucial role in cellular behaviors [75,76,77,78]. Mechanical properties including elastic modulus, tensile strength, and fracture toughness in both macroscopic and microscopic scales, impact cells in a magnitude-dependent manner. Thus, it is vital to maintain optimal mechanical microenvironments to provide a physiological environment accommodatable for cell survival and differentiation [79]. Furthermore, biomechanical signals and the interactions between cells and ECM direct cell specification [80] as stem cell differentiation is highly sensitive to mechanical inputs, especially the stiffness of adherent surfaces [81,82]. Based on the mechano-sensitivity of the cells, the application of mechanical forces or stimulation is emerging as an effective modality to guide cellular behaviors such as proliferation and differentiation, and further form desired tissues under well-controlled tissue morphogenesis.

Mechanically tuned scaffolds can provide a platform to intrinsically (i.e., substrate stiffness) or extrinsically (i.e., applied forces) control mechanical environments to achieve desired cellular responses, as listed in Table 2. The main difference between the two modalities is that intrinsic mechanical modulation is aimed at directly modifying the mechanical properties of scaffolds via control over the substrate’s composition and structure while extrinsic mechanical modulation leverages external mechanical forces to modulate the dynamic mechanical environments of the cells. Intrinsic mechanical control can be achieved by adjusting various properties of polymer scaffolds such as stiffness, viscoelasticity, and structure to affect cellular behaviors via mechanotransduction. For instance, substrate stiffness induces/augments stem cell differentiation toward a specific lineage when it mimics the stiffness of native tissue/ECM of interest by influencing the cytoskeletal organization and subsequent mechano-responsive signaling cascades [83]. Recent studies have focused on designing different scaffold types with specific mechanical properties with mechanical complexities such as stress–strain behavior, viscoelasticity, and stiffness, so as to more closely mimic the native mechanical environment of the target tissue.

Hydrogels are commonly utilized biomaterials to investigate the mechanotransduction behaviors of tissues and cells due to their characteristics of good biocompatibility, effective mass transfer [84], similarity to natural ECM [85], and adjustable stiffness [86]. Bryant et al. entrapped chondrocytes in photo-cross-linkable hydrogel scaffolds based on poly(ethylene glycol) (PEG) with two crosslinking densities, where the hydrogel with the higher density was observed to have 11-fold higher compressive modulus [87]. They found that varied crosslinking densities may lead to different levels of chondrocyte deformation and heterogeneity, resulting in different levels of cartilage ECM regeneration. Sun et al. demonstrated that the stiffness of 3D gelatin hydrogel was highly increased without changing the microstructure of the scaffold when treated with 1-Ethyl-3-[3-dimethylaminopropyl] carbodiimide hydrochloride [88]. Mesenchymal stem cells (MSCs) within a stiffer gelatin hydrogel exhibited a tendency to differentiate to the osteogenic phenotype, leading to greater bone formation.

Furthermore, Rammensee et al. synthesized bis-acrylamide/oligonucleotide polyacrylamide (PAM) hydrogels whose stiffness could be reversibly regulated by controlling the number of DNA hybridization crosslinks [89]. NSCs exhibited greater neurogenesis in the softer hydrogel (0.3 kPa) while neurogenesis was inhibited in the stiffer hydrogel (3 kPa). While these studies well demonstrated the effectiveness of hydrogels as a platform to study the effects of mechanical modulation on cellular behaviors, their applications in vivo are limited. Biocompatibility, differentiation inductivity, stability, and immunomodulating controls are some of the obstacles that need to be addressed before being used therapeutically.

Fibrous scaffolds synthesized by electrospinning have also been widely explored to guide cell proliferation and differentiation since nanofibrous morphology mimics the structure of the native ECM [99]. Depending on the precursor polymer types and electrospinning solution concentrations, fibrous scaffolds with a wide range of stiffness can be fabricated. Similar to the aforementioned hydrogel studies, it has been shown that electrospun scaffold stiffness significantly modulates cell signaling, morphology, and differentiation behaviors. For example, Sack et al. found that endothelial cells cultured on stiff material decreased the β1 integrin activity, leading to the reduction of VEGF internalization and vascular endothelial growth factor receptor 2 (VEGFR2) downregulation, resulting in less angiogenesis [100].

Our research group previously explored the relationship between the mechanical properties of electrospun fibrous substrates and induced pluripotent stem cells (iPSCs) colony morphology [101]. The results showed that iPSCs cultured on softer (19 kPa) electrospun nanofibrous scaffolds exhibited round 3D spherical cell colony morphology whereas stiffer substrate (193 kPa) induced a spread 2D colony morphology. Such a difference in the colony morphology directly influenced the spontaneous differentiation of iPSCs towards ectodermal lineage especially when the cells were cultured on soft material, providing a means to modulate iPSCs’ self-renewal and spontaneous differentiation by manipulating iPSC colony morphology using diverse electrospun substrates having different stiffnesses. Unlike the hydrogel system where stiffness is controlled by modulating the concentration of hydrogel or crosslinking density, electrospun nanofibrous scaffolds provide a means to control scaffold stiffness in a wide range without changing microstructure (thus the availability of adhesion sites) and surface chemistry.

**Figure 2:**
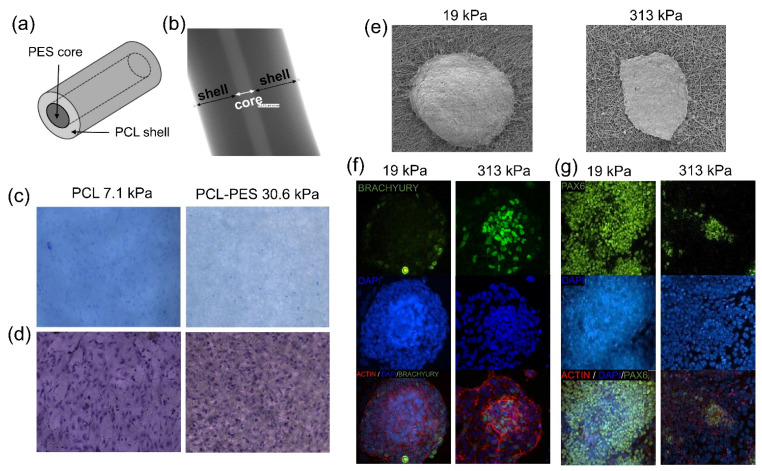
Modulation of stem cell differentiation via scaffold stiffness. (**a**,**b**) A schematic and a TEM image showing a core–shell PES-PCL electrospun nanofiber. (**c**) Alcian blue staining images showing greater chondrogenic differentiation of MSCs cultured on softer pure PCL (left) as compared to stiffer PCL-PES core–shell fibers (right). (**d**) Histological images showing greater alkaline phosphatase activity indicating enhanced osteogenic differentiation of MSCs cultured on stiffer PCL-PES core–shell nanofibers (right) as compared to softer pure PCL (left) [93]. (**e**) scaffold stiffness-dependent induced iPSC colony morphology on soft (left) and stiff (right) nanofibers. (**f**,**g**) Fluorescence images showing stiffness-dependent mesendodermal and ectodermal differentiation of iPSCs [102].

Besides the method of utilizing different polymer materials to control the stiffness of the cell culture scaffolds [102], core–shell electrospinning provides a unique opportunity to control mechanical properties of scaffolds independent of surface chemistry, rendering greater freedom to tailor-design scaffolds for specific applications [103]. For instance, Nam et al. has optimized the electrospinning process and successfully synthesized core–shell polyethersulfone (PES)-PCL fibers with tunable stiffness by controlling the ratio between the two polymers [93]. They further found that nanofibers with higher stiffness (30.6 MPa) supported enhanced osteogenesis while pure PCL with lower stiffness (7.1 MPa) promoted chondrogenesis, demonstrating the impact of the mechanical factor in electrospun scaffolds, decouple from many other factors such as surface chemistry and scaffold morphology, on stem cell differentiation (Figure 2a–d).

Various nanofibers composed of different polymer precursors such as PCL, PES, polycarbonate-urethane, or polyether-ketone-ketone (PEKK) were utilized to examine the relationship between substrate stiffness and the differentiation behavior of iPSCs [102]. The results showed that distinct colony morphologies were observed depending on the scaffold stiffness, which in turn affected the differentiation tendency of stem cells; iPSCs cultured on the stiffer substrate tended to differentiate more towards mesendodermal lineage while more ectodermal differentiation was observed on the softer substrate (Figure 2e–g). Based on these results, the effects of substrate stiffness on the differentiation of iPSCs towards various cell phenotypes throughout various stages were investigated [94]. Results showed that not only the differentiation efficiency of stem cells toward a specific phenotype is significantly affected by substrate stiffness, but the optimal stiffness also dynamically changes during each step of the differentiation process.

Besides the effects of the intrinsic mechanical properties of polymeric scaffolds on tissues and cells, extrinsic mechanical control of tissues and cells has become a promising method to modulate biological responses. The application of external stress or strain requires a scaffold with suitable physical properties such as stiffness and morphology, which also influence cell fate through the activation of different cell signaling pathways. Unlike tensile forces which can be applied to adherent cells on any flexible substrate with a proper surface modification, the application of compressive forces requires a scaffold that provides a 3D microenvironment for appropriate cell viability, proliferation, and differentiation while transferring applied forces to the cells.

The hydrogel system has been the most common platform for such studies as it provides in vivo like microenvironments by encapsulating the cells in a 3D space. Compressive strains with a physiologically relevant magnitude on stem cells encapsulated within agarose or PEG hydrogels have been shown to induce chondrogenesis of stem cells while their effectiveness depended on the degree of lineage specification [104,105]. Koo et al. demonstrated the feasibility of using frequency-shifted (2 MHz to 4 MHz) ultrasound actuation to help form three-dimensional network-structured tissue by aligning fibroblast cells in the alginate hydrogel mixture with polystyrene microparticles [98].

**Figure 3:**
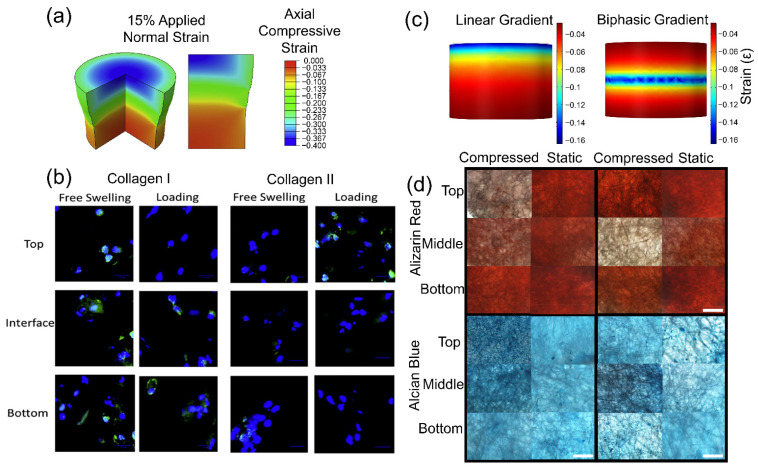
Effect of compressive strain gradient on osteogenic and chondrogenic differentiation. (**a**) A computational model describing a compressive strain gradient through the thickness of multi-layer hydrogel. (**b**) Collagen I and Collagen II expression of cells cultured in the hydrogel with or without applying compressive loading showing differential osteogenic and chondrogenic differentiation levels depending on the magnitude of local compressive strain [106]. (**c**) Computational modeling to design linear or biphasic strain gradient within a monolithic 3D electrospun core–shell nanofibrous scaffold via variable core–shell ratio. (**d**) Histology images of cell/scaffold constructs showing compressive strain gradient-dependent osteogenic (Alizarin red staining) and chondrogenic (Alcian blue staining) differentiation within the individual scaffolds [96].

In addition, Steinmetz et al. developed a hydrogel system having separate layers of different stiffness and demonstrated a compressive strain-dependent MSC fate specification where high compressive strain enhanced chondrogenesis while low compressive loading enhanced osteogenesis (Figure 3a,b) [106]. As mentioned above, however, poor mechanical properties especially for hard tissues, limited range of stiffness control, and cytotoxic effect of leftover crosslinking reagents are several limiting factors for the applications of hydrogels in developing advanced and functional tissue.

Electrospun scaffolds have also been utilized to investigate the effects of applied compressive forces on cellular behaviors. Typical nano-sized electrospun fibers, however, prevent the infiltration of cells into the 3D scaffold, limiting its application in mechanobiology studies. Among various approaches to overcome the limitation, the use of electrospun microfibrous scaffolds provides a means to enable cellular infiltration throughout the appreciable thickness of 3D scaffolds while maintaining mechanical integrities under applied compressive forces. Using the electrospun microfibrous scaffold, externally applied compressive forces have been shown to induce functional maturation in osteoblasts, enhancing ECM secretion by activating SMAD 1/5/8 phosphorylation through type 1 BMP receptor [107]. Another example demonstrated that articular chondrocytes or osteoblasts cultured on microfibrous PCL scaffolds and subjected to dynamic (10% cyclic compressive strain at 1 Hz for 3 h/day) culture conditions expressed anabolic BMPs, applicable to osteochondral tissue engineering [95]. The scaffold was also utilized to demonstrate the magnitude-dependent MSC differentiation toward chondrocyte and osteoblast under compressive loadings, where a high magnitude of compressive loading induced greater chondrogenesis while a low magnitude enhanced osteogenesis, consistent with the results discussed in the above hydrogel culture system [108]. Based on this magnitude-dependent differentiation behavior of MSCs under dynamic compression, a novel core−shell electrospinning method was developed to generate a spatially controlled stiffness gradient in a three-dimensional electrospun scaffold, which presents a strain gradient to the cells inoculated in the scaffold under compressive loading (Figure 3c,d) [96]. Within the monolithic scaffold, the cells in the high strain area differentiated to chondrocytes while osteogenesis was induced in the low strain area, providing an innovative platform to recapitulate the gradient structure for osteochondral regeneration.

Mechanically tuned scaffolds have extensive applications in tissue engineering and regenerative medicine. Numerous in vitro studies showed the great potentials of mechanically tuned scaffolds in directing cellular behaviors, especially guiding stem cell differentiation. However, in vivo studies on mechano-modulation by functional scaffolds are still limited and are of prime importance to exploit their therapeutic potential. Furthermore, the incorporation of other modifications such as biochemical cues into the mechanical control would provide a more robust control over cellular behaviors. Such mechano-stimulatory approaches need to base on a fundamental understanding of the mechanisms of mechano-transduction for the development of tissue-specific scaffolds.

## 4. Magnetic Scaffolds

The application of magnetic fields is another method to modulate cellular behaviors to aid in tissue formation and wound healing. It has been reported that a magnetic field (MF) and/or an electromagnetic field (EMF) play essential roles in determining cell adhesion, migration, and differentiation, thus affecting tissue regeneration and repair [109]. Specifically, pulsed EMFs, in an intensity-dependent manner, have been shown to enhance the wound healing process by modulating cell proliferation, apoptosis, differentiation as well as cell cycles [110,111]. Such pulsed EMFs are produced in a coil when a current is generated by a pulse generator passing through the coil [112]. An example that demonstrates the clinical potential of EMFs is signified by Boopalan et al. [112], where they investigated the efficacy of a pulsed EMF for the treatment of experimental osteochondral defect in a rabbit model. Exposing the osteochondral defect with pulsed EMFs at a frequency of 1 Hz and magnitude of 20 volts for one hour a day for a six-week duration, exhibited the enhanced healing of a full-thickness articular cartilage defect. Another research demonstrating the beneficial effects of applied MFs towards tissue regeneration at the cellular level was conducted by Girolamo et al. [113]. They investigated whether low-frequency pulsed EMFs affect the proliferation and tissue-specific gene expression of human tendon cells as well as the release of appropriate cytokines from those cells. Specifically, the effects of pulsed EMFs with various durations of pulsed EMF stimulation on tendon-specific gene transcription and the release of pro-and anti-inflammatory cytokines of VEGF were investigated. The study demonstrated that pulsed EMFs enhance the proliferation, release of anti-inflammatory cytokines, tendon-specific marker expression, and angiogenic factors in a dose-dependent manner.

Despite these phenomenological observations, the precise molecular mechanisms underlying the effects of pulsed EMFs on cellular behaviors are not fully understood. A recent study suggested that pulsed EMF exposure leads to an increase in cytosolic Ca^2+^ and the activation of calmodulin, which are important factors associated with cell differentiation [114]. However, the activation of ion channels and subsequent signal cascades are believed to be just a fraction of the overall complex cell signaling, which requires extensive investigation to fully understand the influence of MFs on cellular behaviors.

Nevertheless, based on such beneficial effects from magnetic stimulation, the combination of polymeric scaffolds and EMF exposure (Table 3) has gained more research interest recently. 

The utilization of EMFs has shown its feasibility in bone tissue repair. Chen et al. investigated the combinational effect of a sinusoidal EMF and a biochemical factor, VEGF, on the osteogenesis and angiogenesis of MSC-laden PCL/HA implants in a rat subcritical cranial defect. In this study, they seeded rat bone marrow-derived MSCs into PCL/HA composite scaffolds which were either stimulated by VEGF or sinusoidal EMF to construct a vascularized tissue-engineered bone graft [122]. It was found that both the sinusoidal EMF and VEGF could enhance the protein and mRNA expression levels of osteoblast- and endothelial cell-related markers. Furthermore, the combination of the sinusoidal EMF and VEGF synergistically promoted the angiogenic differentiation of MSCs, demonstrating the efficacy of magnetic stimulation by augmenting typical biochemical-mediated controls over cellular behaviors. Similar work by Lajimi et al. demonstrated such a synergistic effect by utilizing electrospun PCL nanofibers along with a pulsed EMF on osteogenic differentiation of iPSCs [123].

In this study, an extremely low frequency pulsed EMF was utilized in combination with PCL nanofibers; it was demonstrated that pulsed EMF alone can induce osteogenic differentiation. However, the differentiation efficiency can be significantly enhanced when combined with cell culturing on the PCL nanofibers. In addition, using a cell type-specific polymeric scaffold along with EMF allows for the promotion of gene expressions that is vital for specific tissue regenerative therapies [124]. These studies demonstrate that the appropriate combination of morphological control by polymeric scaffolds and biophysical control by magnetic stimulation can promote desired cell behaviors and enhance tissue repair.

From what can be inferred from various studies that corroborated the synergistic effects between polymeric scaffolds and the applied MFs, there’s certainly a great potential of magnetic stimulation for clinical applications. However, despite such advantages, there are some drawbacks when it comes to utilizing a magnetic field on cells. It has been shown that when cells are subjected to a magnetic field of 4 tesla or greater, there’s a possibility for physiological and growth abnormalities at the cellular level [125]. In that case, it is important to account for the intensity of the magnetic fields being used for tissue engineering. One approach to avoid the harmful effects of strong magnetic exposure is to integrate magnetic nanoparticles (MNPs) into polymeric scaffolds, allowing the use of magnetic fields in lower magnitudes due to the proximity of the magnetic origin to the cells. Among various types of MNPs, iron and iron oxide are the most commonly used MNPs to produce polymer/MNP composites [126].

Various fabrication methods have been used to incorporate MNPs into the polymeric network of the scaffold in order to produce magnetic scaffolds [127]. One method utilizes incorporating MNPs into a scaffold network through diffusion [116]. Sangram et al. fabricated biomimetic magnetic silk scaffolds by infiltrating iron oxide MNPs to the matrix through a diffusion process. This process employed the use of MNPs and bioagent-conjugated MNPs (growth factors, and other proteins) in porous interconnected silk scaffolds. The diffusion process was facilitated by the application of a magnetic field with varying intensities, successfully integrating the MNPs into the scaffold network [116]. Another common fabrication method uses a simple mixing of a polymer solution with MNPs before structuring scaffolds. Kim et al. utilized iron oxide magnetic nanorods (MNRs) to create a magneto-responsive polymeric scaffold [128]. Dispersion of magnetic nanorods in the polymer solution was key for the successful and uniform integration of magnetic particles into the polymer scaffold. Similarly, Moradian et al. developed PCL scaffolds containing 3 wt.% of relatively uniformly distributed cobalt-zinc ferrite nanoparticles (CZF-NPs) by electrospinning a mixture of PCL solution and CZF-NPs [129].

Since MNPs exhibit their own magnetic microenvironment, encapsulating them in a polymeric scaffold can promote the proliferation of the adherent cells and enhance their cellular activities. In the study done by Shuai et al., how the magnetic micro-environment from Fe_3_O_4_/MNPs affects bone regeneration was investigated [120]. A polymeric scaffold using PLLA/polyglycolic acid (PLLA/PGA) via selective laser sintering was utilized to investigate the degree of bone regeneration depending on different concentrations of MNPs encapsulated within the scaffold. An in vivo study further demonstrated that the capability of the local magnetic fields from the scaffolds to accelerate bone regeneration as well as to enhance the compressive strength and modulus of the scaffolds. Another prime example that demonstrates MNP’s capability to provide a microenvironment to enhance tissue regeneration can be found in the work of Kim et al. [121]. They fabricated magnetic scaffolds composed of PCL and functionalized magnetite nanoparticles and characterized their physicochemical, mechanical, and biological properties for effective bone regeneration. Magnetite (Fe_3_O_4_) nanoparticles were surface-functionalized and encapsulated into a PCL polymeric scaffold. The MNPs incorporated into PCL scaffolds were demonstrated to promote the mineral formation and stimulate cellular adhesion while exhibiting good tissue compatibility. These examples showcase the anabolic effects of MNPs when incorporated within a polymeric scaffold, even without an applied external magnetic field. The cytotoxicity of the MNPs, however, is still a major challenge that needs to be addressed to prevent any adverse immune response from occurring towards the host. With that addressed, MNPs incorporated polymeric scaffolds will have a great potential in future diagnostic and clinical applications.

## 5. Exogeneous Activation of Multi-Functional Scaffolds

### 5.1. Magneto-Responsive Scaffolds

As described above, both mechanical and magnetic stimulations have been shown to modulate cellular behaviors including migration, proliferation, and differentiation. In addition to the utilization of individual stimuli, the activation of magneto-responsive polymeric scaffolds via the exogeneous application of EMFs has been recently introduced in the field of tissue engineering. Instead of utilizing magnetic fields or mechanical stimulation alone to stimulate tissues and cells, magneto-responsive polymeric scaffolds by encapsulating MNPs into polymeric scaffolds provide an opportunity to induce mechanical perturbation under the applied magnetic fields [130]. The high-frequency vibration of MNPs in the polymeric scaffolds in a dynamically varying magnetic field would mechanically deform the substrate and stimulate adherent cells in the nano or microscale. Such a combination of magnetic and mechanical stimulation will likely influence a series of cellular behaviors including activation of magnetic and mechanical sensitive channels, cytoskeleton reorganization, and expression of specific genes, resulting in a more controllable and accurate physical stimulation [131].

Magneto-responsive polymeric scaffolds under the applied magnetic fields have been shown to improve cellular behaviors and used for a wide variety of tissue engineering applications, as listed in Table 3. For example, Reizabal et al. electrospun silk fibroin (SF) nanofibrous scaffolds, embedded with 0–20% of cobalt ferrite (COF) magnetic particles [132]. They further demonstrated that mechanical stimulation generated by the SF/COF composite scaffold under the dynamic application of magnetic fields significantly enhanced the cell viability and induced a favorable cell morphology for proliferation. Similarly, Abdeen et al. synthesized a magneto-responsive hydrogel which was formed by embedding carbonyl iron particles in a PAM hydrogel matrix [117]. They utilized the application of a magnetic field in various magnitudes and polarities to reversibly control the stiffness of the hydrogel. Under the stimulation of both applied magnetic field and magnetic field-induced stiffness change, MSCs exhibited enhanced cell spreading. The potential of angiogenesis and osteogenesis was further observed, providing a means of utilizing the applied magnetic fields to efficiently control the differentiation of MSCs for angiogenesis and osteogenesis.

In addition, Goncalves et al. utilized iron oxide MNPs and embedded them in electrospun PCL fibers for a tendon tissue engineering application [115]. By applying a constant magnetic field of 0.35 T for 7 days, they showed the activation of mechano-sensitive ion channels and the subsequent tenogenic differentiation of adipose tissue-derived MSCs, based on the enhanced synthesis of tenascin C and collagen type I rich matrix from the cells under the applied magnetic fields. For the application of magneto-responsive polymeric scaffolds in nerve tissue engineering, Liu et al. fabricated a nanocomposite scaffold composed of MNPs and a biodegradable chitosan-glycerophosphate polymer [119]. Tunable magnetization, and degradation rate as well as the maintenance of Schwann cell viability after transplantation were demonstrated under a magnetic field, potentially suggesting the synergistic effects of magnetic and mechanical stimulation.

Magneto-responsive polymeric scaffolds have demonstrated excellent potential for various biomedical applications. An advantage of using such scaffolds includes the controllable conformational and chemical environment changes that occur within the scaffolds in response to a magnetic field. These changes have been shown to not only change the mechanical properties of the polymeric scaffolds through magnetic particles vibration and polymer deformation [133], but also lead to the release of therapeutic agents embedded within the scaffolds with more desirable pharmacokinetics [134].

However, there are several limitations to be addressed for the facile adoption of magneto-responsive scaffolds in clinical applications. One of the major disadvantages in the in vivo application of MNPs includes their low biocompatibility and biodegradability in the physiological medium [135]. Another disadvantage of using MNPs is their low colloidal stability and the tendency to agglomerate [136]. To overcome these limitations, surface modification by coating with organic and inorganic species is typically employed [136]. In order for the interaction between cells and polymer/MNP composites to be beneficial, it’s important to take into consideration the cell type that is being used, the surface modification to be applied to the MNPs, the cell medium composition as well as the nanoparticle interaction and oxidation state of the magnetic elements [137]. By carefully designing polymer/MNP composites based on the consideration of these parameters, the polymeric scaffold is less likely to have any cytotoxic effects as compared to the raw form of MNPs.

### 5.2. Piezoelectric Polymeric Scaffolds

Piezoelectric materials have been well studied in a diverse research field for their ability to interconvert energies between electrical and mechanical origins. The direct piezoelectric effect, first discovered by French physicists Jacques and Pierre Curie, is that materials generate an electrical potential signal under mechanical stress, whereas the conversion from electric energy to mechanical energy is called the reverse piezoelectric effect [138,139]. Governed by these direct and indirect piezoelectric effects, piezoelectricity has been exploited in a variety of applications in areas of energy, healthcare, and environment including sensors, drug delivery, filtration, electrode materials for batteries, supercapacitors, fuel cells, and solar cells, catalytic support, and smart textiles as well as a scaffold for tissue engineering [16,17,140]. Interestingly, mammalian tissues including bone, cartilage, ligaments, skin, and tendons exhibit piezoelectricity [17,141,142,143,144,145]. In these tissues, collagen is the key component for their piezoelectricity where the natural helical structure of polymer chains within the collagen enables its hydrogen bonds to create aligned dipoles that can respond to an external electrical field or shear force to produce the shear piezoelectric effect [17,146]. The shear piezoelectric coefficient of collagen is reported to be approximately 2–3 pC/N [147]. Due to this piezoelectrical property, electrical signaling or action potentials can be activated in response to internal mechanical forces; voltage-gated channels existing on cellular membranes will detect and respond to these electrical signaling and activate downstream signaling pathways that regulate various cellular behaviors including proliferation, migration, differentiation, and maturation [148]. Therefore, there is an increasing effort to utilize either natural or synthetic piezoelectric materials to control and regulate cellular behaviors [16,17,148].

The magnitude of the piezoelectric effect in a material depends on the material’s crystal structure. Inorganic piezoelectric materials such as barium titanate (BaTiO_3_), zinc oxide (ZnO), and lead zirconate titanate (PZT) usually exhibit greater piezoelectric responses due to their superior periodicity in the crystal structure [27,149,150]. Despite their excellent electromechanical properties; however, these inorganic materials are brittle, therefore limiting the applications in the biological field, which usually requires a relatively low stiffness to avoid a mechanical mismatch with native tissues [151]. In contrast, organic (polymeric) piezoelectric materials are mechanically flexible, providing an alternative suitable for a low frequency and high strain mechano-biological environment [152]. Table 4 lists some of the characteristics of the most popular piezoelectric polymeric scaffolds and their biological results.

PVDF and its copolymers poly(vinylidene fluoride-trifluoroethylene (P(VDF-TrFE)), are by far the most well studied polymeric piezoelectric materials due to their excellent transverse piezoelectric effect [159,160]. PVDF normally possesses chain conformation of trans (T) and gauche (G) linkages (i.e., TGTG’), which constitutes thermodynamically stable α-phase at the ambient temperature. In order to exhibit the piezoelectric effect, the polymer chains of PVDF need to be rearranged to contain all-trans conformation (i.e., TTTT) or conformation of (T3GT3G’) that is β-phase or γ-phase, respectively. The unidirectional reorientation of β-phase under physical stresses, i.e., mechanical stretching, results in a net dipole development perpendicular to the direction of the stress. Researchers have developed various techniques and methods to enhance the piezoelectric response of PVDF and its derivatives P(VDF-TrFE) [161,162,163].

Electrospinning is one of the most commonly used techniques to produce PVDF nanofibrous scaffolds with high piezoelectricity [162]; by intrinsically applying a high voltage field to the polymers during the electrospinning process, polymer domains and chains are aligned unidirectionally to increase the formation of overall electroactive phases. In addition, electrospinning has also been shown to mechanically pull the fibers due to the Taylor cone stretching and elongating during the process, further improving piezoelectricity [159]. Chowdhury et al. compared the values of *d*_33_, a piezoelectric coefficient describing how efficiently the material can convert electrical energy to mechanical energy [164]. It was found that electrospun PVDF fiber having a fiber diameter of 105 nm has a significantly higher *d*_33_ value (32 pC/N) as compared to that of PVDF pellet (5 pC/N), demonstrating the potential of electrospinning on the enhancement of the piezoelectric property. To further enhance the piezoelectric response of PVDF and its copolymers, multiple approaches have been utilized to optimize the electrospinning process and/or post-treat the electrospun nanofibers, including controlling fiber diameter and thermal treatment [151,165].

We recently showed that there was a substantial increase in *d*_33_ value from 20 pC/N to 56 pC/N when the fiber diameter of electrospun P(VDF-TrFE) decreased from 500 nm to 30 nm [151], likely because of fiber diameter reduction leading to an overall increase in crystallinity structure in polymer and resulting in an increase in electroactive β-phase content. Furthermore, it was also demonstrated that 90 °C thermal treatment significantly enhances the piezoelectric property, where the *d*_33_ value of the thermally treated electrospun P(VDF-TrFE) nanofibers having 30 nm fiber diameter reached up to 108 pC/N, comparable to those values exhibited in inorganic piezoelectric materials [165]. Phase analysis indicated that the significant enhancement of piezoelectric properties was highly attributed to the increase of the electroactive β-phase under the synergistic effect of dimensional reduction and phase re-organization.

Besides its excellent piezoelectricity, P(VDF-TrFE) shows great biocompatibility which enables the use of P(VDF-TrFE) in the tissue engineering field. So far, PVDF and its copolymers P(VDF-TrFE) have been utilized to induce or enhance the differentiation behavior of stem cells including osteogenesis, chondrogenesis, and neurogenesis. Damaraju et al. utilized heat-treated electrospun P(VDF-TrFE) scaffolds to culture MSCs which showed an increase in both osteogenesis and chondrogenesis as compared to those cells cultured on non-piezo PCL scaffolds [154]. Interestingly, they also found that cell lineage differentiation was dependent on the level of piezoelectric properties where low piezoelectric P(VDF-TrFE) scaffold enhanced more towards chondrogenesis while higher piezoelectric P(VDF-TrFE), whose piezoresponse was improved by heat treatment, induced osteogenic differentiation. Similarly, Lee et al. compared the differentiation behavior of human NSCs cultured on as-spun (less piezoelectric) or annealed electrospun (more piezoelectric) aligned P(VDF-TrFE) fibrous scaffolds [166]. The results showed that annealed P(VDF-TrFE) scaffolds promoted the formation of mature β3 tubulin-positive neuronal cells and had a longer neurite extension as compared to the cells cultured on as-spun scaffolds.

Poly(3-hydroxybutyrate-co-3-hydroxyvalerate) (PHBV) has been recently utilized in bone and cartilage tissue engineering due to its similar piezoelectric coefficients (~0.8 pC/N) to the native collagen that constitutes native bones and cartilages. Gorodzha et al. compared the cellular behaviors of MSCs cultured on electrospun piezoelectric PHBV scaffolds and electrospun non-piezoelectric PCL scaffolds. They found that there was greater calcium deposition on PHBV scaffolds as compared to PCL scaffolds due to the minor shear piezoelectricity of PHBV [158]. For cartilage, Kose et al. utilized porous PHBV scaffolds to culture chondrocytes, and the cell/scaffold constructs led to a full repair of cartilage defects in vivo [157]. In addition, several studies synthesized PHBV as a composite with other materials that possess greater piezoelectric properties to compensate for the low piezoelectric effect of PHBV itself. Jiao et al. showed an improved piezoelectric coefficient of PHBV/Barium titanate (PHBV/BT) composite up to 1.5 pC/N depending on the amount of BT added [167]. Similarly, Gorodha et al. successfully synthesized PHBV/silica HA (PHBV/SiHA) composite having a piezoelectric coefficient of 1.56 pC/N, which is probably attributed to natural piezoelectric properties of stoichiometric HA [158].

PLLA has recently gained significant research interest for its unique, excellent shear piezoelectric property. PLLA normally exhibits thermodynamically stable conformations of α and α’ phases, where the CO-O- dipoles are helically oriented along the main backbone chain [168]. Polarization of the chain molecules is induced when the helical conformation structure is sheared along its side chains, resulting in the charge separation parallel to the plane of applied shear stress [168]. PLLA has been previously shown to exhibit the highest value of the shear piezoelectric coefficient of *d*_14_ at approximately 12 pC/N [169]. We recently found that the shear piezoelectric property of electrospun PLLA nanofibers can be further tuned by annealing the samples using different temperature regimens [155]. When the annealing temperature was above the glass transition temperature of PLLA (65 °C), the shear piezoelectricity was significantly improved due to the increase in the electroactive α’ phase. However, further increase of annealing temperature above 110 °C resulted in a reduction of the shear piezoelectricity due to a decrease in the α’ phase content in the electrospun PLLA nanofibers. Moreover, it was also found that the electrospun PLLA nanofibers possess the orthogonal piezoelectric property, similar to P(VDF-TrFE) nanofibers, probably due to the high electric field poling during electrospinning as discussed earlier. The orthogonal piezoelectric property could be improved by decreasing the fiber diameter due to the enhanced alignment of polymer chains. More interestingly, the annealing temperature above the glass transition point almost eliminated the orthogonal piezoelectric effect from electrospun PLLA nanofibers by decreasing the amorphous electrospun phase. This flexible modulation of orthogonal and shear piezoelectric properties provides a means for the diverse applications of PLLA. Specifically, the biocompatibility and biodegradability of PLLA enable it to be applied to a broader tissue engineering field as compared to other synthetic polymers including PVDF and its derivatives. More polarized surface, greater protein absorption, and better cellular adhesion, proliferation, migration, and differentiation were often observed due to the piezoelectric property of PLLA.

Barroca et al. discovered that neuroblastoma cells cultured on electrospun aligned polarized PLLA nanofibers showed higher differentiation efficiency [170]. They also found that cortical neurons cultivated on poled PLLA nanofibers showed increased neurite outgrowth up to approximately 330 μm as compared to 200 μm of the control group where cells were cultured on the regular coverslips. For an in vivo application, Fukada, et al. demonstrated an enhanced bone regeneration by a PLLA scaffold, which had a shear piezoelectric coefficient of 10 pC/N, possibly due to the ionic current activation of bone cells by the piezoelectric effect [156].

**Figure 4:**
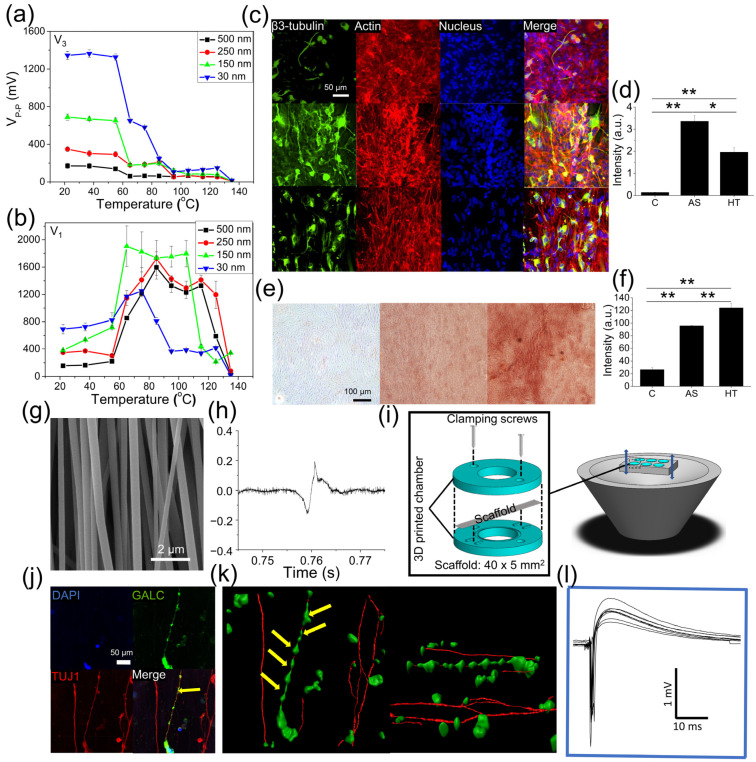
Utilization of piezoelectric polymeric scaffolds for guided stem cell differentiation. (**a**,**b**) Electric outputs of electrospun PLLA nanofibers with various fiber diameters and heat treatment regimens in the (**a**) transverse direction or (**b**) longitudinal direction. (**c**,**d**) Immunofluorescence images of human NSCs cultured on ((**c**), top) tissue culture plate, ((**c**), middle) as-spun, or ((**c**), bottom) 65 °C heat-treated PLLA nanofibers for 1 week showing different degrees of neuronal differentiation, quantified by the intensity of neuronal β3-tubulin expression. (**e**,**f**) Histological images of human MSCs cultured on ((**e**), left) tissue culture plate, (**e**, middle) as-spun, or (**e**, right) 65 °C heat-treated PLLA nanofibers for 2 weeks showing different degrees of osteogenic differentiation, quantified by the intensity of osteogenic calcium deposition via Alizarin red staining [155]. (* and ** denote statistical significance of *p* < 0.05 and *p* < 0.01, respectively.) (**g**) an SEM image of electrospun-aligned P(VDF-TrFE) nanofibrous scaffold and (**h**) its voltage output under 0.03% of strain. (**i**) A schematic showing a cell culture system for the acoustic activation of the piezoelectric P(VDF-TrFE) scaffolds. (**j**) Representative fluorescent images and (**k**) their Imaris 3D reconstruction showing neuron-oligodendrocytic interaction. (**l**) A representative graph showing action potentials generated from neurons derived from NSCs by mechano-electrical stimulation [153].

In a recent study, we systematically examined the differentiation behaviors of human NSCs and MSCs cultured on the electrospun PLLA scaffolds with either high orthogonal piezoelectricity or high shear piezoelectricity, depending on the annealing temperature as described earlier [155]. A significant difference in cell differentiation efficiency was observed where NSCs cultured on high orthogonal piezoelectric PLLA scaffolds exhibited greater neuronal differentiation as compared to those cells cultured on high shear piezoelectric PLLA scaffolds (Figure 4a–f). In contrast, hMSCs showed a greater osteogenic differentiation tendency when they were cultured on high shear piezoelectric PLLA scaffolds. These self-powered piezoelectric stem cell culture platforms provide an opportunity to match the in vivo physiological microenvironment where neurons are subjected to their innate surface potential alteration while MSCs experience shear piezoelectricity originated from collagen aligned with the longitudinal direction of the long bone [157].

Although these studies showed promising results demonstrating the positive effects of piezoelectric polymers on cellular behaviors, the static culture of cells on piezoelectric scaffolds does not take advantage of the full potential of the piezoelectric effect. It is likely that a minimal electric potential is generated under static conditions, as compared to those previously mentioned in the studies which utilized the direct application of external electrical stimulation. To address this limitation, several studies combined both mechanical perturbation and electrical potentials as stimuli to regulate cell behaviors, by taking advantage of the ability of piezoelectric material to convert mechanical energy to electrical energy without the need of external wiring and electrical power supply.

We recently developed a cell culture system where acoustic actuation was used to activate electrospun aligned P(VDF-TrFE) nanofibrous scaffolds to generate electrical potentials (Figure 4g–l) [153]. In this system, both mechanical stimulation and electrical stimulation derived from the piezoelectric activation of the scaffold by acoustic actuation were applied to the cells cultured on the surface of the piezoelectric scaffolds. Acoustic stimulation, causing a 0.03% strain of the electrospun P(VDF-TrFE) nanofibrous scaffolds, was applied to produce −100 mV potentials to human NSCs, resulting in (1) the differentiation of the cells simultaneously towards neurons, oligodendrocytes, and astrocytes; (2) the formation of myelin in a three-dimensional, self-organized neuron-glial interface; (3) the cellular interactions among the different cell populations within this organized 3D structure, leading to superior neural functionality.

This study demonstrates that the activation of piezoelectric scaffolds by exogeneous mechanical stimulation leads to more significant prolonged effects on tissues and cells as compared to static piezoelectric cell culture platforms. The development of highly efficient piezoelectric materials and/or activation methods applicable for in vivo applications will further the application of piezoelectric scaffolds in various tissue engineering fields including bone regeneration, wound healing, and angiogenesis.

## 6. Conclusions

In addition to their role in structural support, polymeric scaffolds with a variety of physical functionality have gained significant research interest in the past decade to regulate cellular behaviors and direct tissue functions in vitro by controlling the physical microenvironments of the cells. The recent development of multifunctional polymeric scaffolds, in combination with exogenously applied stimuli including electrical, mechanical, and magnetic stimulation, has provided novel tools to guide tissue morphogenesis such as the development of the functional neuronal network, effective bone regeneration, and blood vessel formation.

These promising results are expected to lead to the development of functionally mature engineered tissues in vitro for tissue repair implantation, drug discovery platforms, or other diagnostic applications. Such multifunctional polymeric scaffolds have demonstrated anabolic effects on the functional development of tissues and cells. However, there is still a lack of systematic evaluation and control of those physical factors to precisely direct cellular behaviors, resulting in inconsistent or even contradictory results among the studies. Therefore, a more systematic approach needs to be taken to fully understand the effects of various parameters, including magnitude, duration, and frequency of each physical factor, on cell/tissue development. Nevertheless, recent advances in multifunctional polymeric scaffolds are expected to pave the way for efficient tissue engineering strategies for clinical applications.

## Figures and Tables

**Table 1:** Conductive polymers and their tissue engineering applications.

Functional Type	Material	Synthesis Method	Material Properties	External Stimuli	Cell Types	Biological Effect	Reference
Conductive polymers	Poly(L-lactic acid) (PLLA)/multi-walled carbon nanotube (MWNT)	Electrospinning	Conductivity 6 mS/cm	NA	Mouse Embryonic Stem cells (ESCs)	Promoted growth and neural differentiation of mouse ESCs	Kabiri et al. [44]
	MWNT	Glass deposition	Conductivity 3.82 × 10^5^ S/cm	NA	Neonatal rat ventricular myocytes	Enhanced cardiomyocyte growth, proliferation, and maturation	Martinelli et al. [45,46]
	poly(ε-caprolactone)(PCL)/carbon nanotubes (CNT)	Electrospinning	Conductivity 5–35 mS/cm	Electrical stimulation (ES): 10 min/day for 4 days, 500 V/m, and 5 ms pulse width at 1 Hz.	Human MSCs	Enhanced cardiac differentiation of human MSCs on the conductive scaffold without ES and on the nonconductive scaffold with ES	Crowder et al. [47]
	CNT/collagen	Glass deposition	Conductance 1.72 × 10^−9^ Ω^−1^	NA	Neonatal rat ventricular myocytes	Enhanced cardiomyocyte adhesion and maturation.	Sun et al. [48]
	Xanthan/Polypyrrole (PPy)	Electro polymerization	NA	NA	Human fibroblasts	Enhanced cell adhesion and proliferation	Bueno et al. [49]
	PPy/PCL/poly(lactic-co-glycolic acid) (PLGA)/Mg	Photopolymerization	Conductivity around 50 S/m	NA	Human kidney cells	Supported cell growth and proliferation with enhanced biodegradability	Liu et al. [50]
	PPy/Poly(DL-lactide) (PDLLA)	Emulsion polymerization	Resistivity 1 × 10^3^ Ω/square	1000 h with 100 mV DC current density 0–106.67 μA/mm^2^.	Human skin fibroblasts	Improved growth of fibroblasts	Shi et al. [51]
	PPy/chitosan	Microemulsion polymerization	Conductivity 10^−3^ S/cm	100 mV/mm, 4 h	Rat Schwann cells	Supported cell adhesion, spreading, and proliferation with or without ES.	Huang et al. [52]
	PLLA/PPy/Heparin	Solvent casting	Resistivity 5 × 10^3^ Ω/square	200 mV/mm three 6-h periods for 6 days	Osteoblast-like Saos-2 cells	Promoted osteoblast adhesion and growth, cultured on electrically stimulated membranes.	Meng et al. [53]
	Polyaniline (PANi)/Gelatin	Electrospinning	Conductivity 0.01–0.02 S/cm	NA	Rat cardiac myoblast cells	Supported cardiac myoblast cell attachment and proliferation	Li et al. [54]
	PANi/PLLA	Electrospinning	Conductance 3 × 10^−9^ S	100 mV/mm for a period of 60 min	Rat NSCs	Elevated cell proliferation and neurite outgrowth	Prabhakaran et al. [40]
	poly(3,4-ethylenedioxythiophene) (PEDOT)/PLLA	Melt spinning	Resistivity 100 Ω/square	NA	Human skin fibroblasts	Improved cell migration, adhesion, and proliferation	Niu et al. [55]

**Table 2:** Mechanically tuned polymers and their tissue engineering applications.

Functional Type	Material	Synthesis Method	Material Properties	External Stimuli	Cell Types	Biological Effect	Reference
Mechanically tuned polymers	Polyethylene glycol diacrylate (PEGDA)	Photo crosslinking	Compressive modulus (670 ± 120 kPa, 60 ± 3 kPa)	Static compressive strains from 0% to 20%	Chondrocytes	Compressive strain-dependent chondrocyte morphology	Bryant et al. [87]
	PEGDA	Hydrogel 3D printing	Increasing porosity related with decreased sound speed and elastic moduli	10 MHz of ultrasound pulses	Human MSCs	Ultrasound magnitude-dependent cell adhesion and proliferation behavior	Aliabouzar et al. [90]
	Gelatin	Chemical crosslinking with 1-ethyl-3-(3-dimethylaminopropyl) carbodiimide hydrochloride	Substrate stiffness (0.6 kPa to 2.5 kPa)	NA	MSCs	Stifness-dependent osteogenesis	Sun et al. [88]
	Gelatin-methacryloyl (GelMA)	Photo-crosslinking	NA	Surface acoustic wave (a desired frequency with input power from −7 dBm to −12 dBm)	Cardiac cells	Improved cell distribution and enhanced cell viability and functionality	Naseer et al. [91]
	PCL	Electrospinning	Young’s modulus (3D cellular scaffold 136.45 ± 9.15 kPa compared to acellular scaffolds 24.55 ± 8.5 kPa)	10% compressive strain (11.81 ± 0.42 kPa)	Osteoblasts	Induced osteogenesis and enhanced extracellular matrix (ECM) formation by compressive forces.	Rath et al. [92]
	Polyethersulfone (PES)/PCL	Core-shell electrospinning	Substrate stiffness (PES-PCL 30.6 MPa, PCL 7.1 MPa)	NA	Murine embryonic mesenchymal progenitor cells	Stiffness-dependent osteogenesis and chondrogenesis	Nam et al. [93]
	PCL/polyetherketoneketone (PEKK)	Electrospinning	Substrate stiffness (PCL 20 kPa, PEKK 300 kPa)	NA	iPSCs	Stifness-dependent lineage- and developmental stage-specific differentiaion of iPSC colonies	Maldonado et al. [94]
	PCL	Electrospinning	Compressive modulus (710 kPa of dynamic culture vs. 280 kPa of static culture)	10% compressive strain at 1 Hz for 3 h/day, 2 weeks total)	Articular chondrocytes or osteoblasts	Induced osteogenesis by biomechanical stimulation	Nam et al. [95]
	Poly(ethylene glycol) (PEG)-PCL	Core-shell electrospinning	Linear or biphasic mechanical gradient (3 kPa to 19 kPa)	Dynamic compressive loading at a frequency of 1 Hz for 2 h daily for 42 days	Human MSCs	Local strain magnitude-dependent osteogenesis and chondrogenesis	Horner et al. [96]
	Polyacrylamide (PAM)	DNA/Bind-Silane crosslinking	ECM stiffness pulses (70–75 kPa)	NA	Human NSCs	Stiffness-dependent NSCs differentiation	Rammensee et al. [89]
	Poly(N-isopropylacrylamide) (PNIPAm)	Cryo-polymerization crosslinking	Elastic modulus (280 kPa to 20 kPa, then 36 kPa)	NA	Bovine fetal fibroblasts	Prolonged cell growth and proliferation for 70 culture days	Rivero et al. [97]
	Alginate	Chemical crosslinking	NA	Frequency-shifted (2 MHz to 4 MHz) ultrasound actuation	Fibroblast cells	Enhanced cell viability and induced 3D tissue formation	Koo et al. [98]

**Table 3:** Magnetic and magneto-responsive polymers and their tissue engineering applications.

Functional Type	Material	Synthesis Method	Material Properties	External Stimuli	Cell Types	Biological Effect	Reference
Magnetic and magneto responsive polymers	Starch/PCL/Fe_3_O_4_ Magnetic nanoparticles (MNPs)	Rapid Prototyping	Parallel fiber alignment	Magnetic Field (MF) intensity: 0–5 T	Human adipose-derived stem cells	1. Cells undergo tenogenic differentiation synthesizing a Tenascin C and Collagen type I rich matrix 2. Promoted cellular differentiation	Goncalves et al. [115]
	Silk Fibroin Protein/Fe_3_O_4_ MNPs	Lyophilization	N/A	MF Frequency: 293 kHz MF intensity (alternating): 30 mT	Mouse calvaria preosteoblast cells	1. Improved cell adhesion and proliferation 2. Improved colonization of osteogenic cells	Samal et al. [116]
	PAM/Carbonyl Iron particles	N/A	Stiffness 0.12–75 kPa	MF intensity: 0.75 T	Human MSCs	1. Secretion of proangiogenic molecules 2. Dynamic control of osteogenesis	Abdeen et al. [117]
	Polyvinylidene fluoride (PVDF)/CoFe_2_O_4_ MNPs	Solvent Casting	N/A	MF intensity: 0–200 Oe	MC3T3-E1 preosteoblast cells	1. Promote the proliferation of preosteoblasts 2. Increased cell viability	Fernandes et al. [118]
	Chitosan-glycerophosphate/Fe_3_O_4_ MNPs	Lyophilization	N/A	MF Frequency: 0–100 Hz MF intensity: 0–200 mT	Schwann cells	1. Promoted Schwann cell viability after transplantation	Liu et al. [119]
	PLLA/Polyglycolide (PGA)/Fe_3_O_4_ MNPs	Selective layer sintering	N/A	N/A	MG63 cells	1. Stimulated cell adhesion and viability 2. Enhanced proliferation rate and alkaline phosphatase activity	Shuai et al. [120]
	PCL/Fe_3_O_4_ MNPs	Lyophilization	Elastic Modulus (5 wt% MNPs): 1.4 MPa Elastic Modulus (10 wt% MNPs): 2.4 MPa	N/A	MC3T3-E1 cells	1. Increased cell adhesion 2. Increased cellular proliferation confluence 3. Cell mineralization was enhanced 4. Enhanced substantial fibroblastic cell invasion and neo blood vessel formation	Kim et al. [121]
	PCL + hydroxyapatite (HA)	3D Bioprinting	N/A	Sinusoidal MF Intensity: 1 mT	rat bone marrow-derived MSCs	1. Enhanced the protein and mRNA expression levels of osteoblast- and endothelial cell-related markers 2. Promoted the angiogenic differentiation of rBMSCs 3. Proteins of Wnt1, low-density lipoprotein receptor-related protein 6, and β-catenin increased in all inducted group	Chen et al. [122]
	PCL	Electrospinning	N/A	MF Frequency: 50 Hz Pulsed MF Intensity: 1 mT	Human iPSCs	Increased in iPSC differentiation into an osteogenic lineage	Ardeshirylajimi et al. [123]

**Table 4:** Piezoelectric polymers and their tissue engineering applications.

Figure	Material	Synthesis Method	Material Properties	External Stimuli	Cell Types	Biological Effect	Reference
Piezoelectric polymers	poly(vinylidene fluoride-trifluoroethylene (P(VDF-TrFE))	Electrospinning	*d*_33_ = 24 pC/N	Mechanical strain: 12 HZ 0.03% Electric output: –100 mV	Human NSCs, mouse NSCs	1. Multi-phenotypic differentiation of cells towards neurons, oligodendrocytes and astrocytes. 2. Induction of myelination. 3. Functional neuronal network development	Tai et al. [153]
	P(VDF-TrFE)	Electrospinning	NA	10% compressive strain 1 HZ Electrical output: 100 mV	Human MSCs	Piezoelectric property-dependent induced chondrogenesis and osteogenesis.	Damaraju et al. [154]
	PLLA	Electrospinning	*d*_33_ = 4.7 pC/N	NA	Human NSCs, Human MSCs	Piezoelectric property-dependent neurogenesis and osteogenesis	Tai et al. [155]
	PLLA	Solvent casting	*d*_14_ = 10 pC/N	NA	Cat tibia and fibula implantation	Enhanced bone regeneration and growth	Fukada et al. [156]
	Poly(3-hydroxybutyrate-co-3-hydroxyvalerate) (PHBV)	Solvent casting	NA	NA	Rabbit chondrocytes	Improved cartilage healing in vivo using chondrocytes seeded PHBV where new cartilage formation was observed	Köse et al. [157]
	PHBV/silicate/HA	Electrospinning	*d*_33_ = 1.558 pC/N	NA	Human MSCs	Promoted cell adhesions, osteogenic differentiation.	Gorodzha et al. [158]

## Data Availability

Not applicable.

## Abbreviations

AP	alkaline phosphatase
BaTiO_3_	barium titanate
BMP	bone morphogenetic protein
CNT	carbon nanotubes
CPs	conductive polymers
CZF-NPs	cobalt-zinc ferrite nanoparticles
ECM	extracellular matrix
ECs	endothelial cells
EMF	electromagnetic field
ESCs	embryonic stem cells
Fe_3_O_4_	ion oxide
GelMA	gelatin-methacryloyl
HA	hydroxyapatite
HE	heparin
iPSCs	induced pluripotent stem cells
MF	magnetic field
MNPs	magnetic nanoparticles
MNRs	magnetic nanorods
MSCs	mesenchymal stem cells
MWNT	multi-walled carbon nanotube
NSCs	neural stem cells
P(VDF-TrFE)	poly(vinylidene fluoride-trifluoroethylene
PAM	polyacrylamide
PANi	polyaniline
PCL	poly(ε-caprolactone)
PDLLA	Poly(DL-lactic acid)
PEDOT	poly(3, 4-ethylenedioxythiophene)
PEG	poly(ethylene glycol)
PEGDA	polyethylene glycol diacrylate
PEKK	polyetherketoneketone
PES	polyethersulfone
PGA	polyglycolide
PHBV	poly(3-hydroxybutyrate-co-3-hydroxyvalerate)
PLA	poly(lactic acid)
PLGA	poly(lactic-co-glycolic acid)
PLLA	poly(L-lactic acid)
PNIPAm	poly(N-isopropylacrylamide)
Ppy	polypyrrole
PSS	polystyrene sulfonate
PVDF	polyvinylidene fluoride
PZT	lead zirconate titanate
Runx-2	runt related transcription factor 2
SWNT	single-walled carbon nanotube
VEGF	vascular endothelial growth factor
VEGFR2	vascular endothelial growth factor receptor 2
ZnO	zinc oxide
